# Personalized medicine to treat refractory benign paroxysmal positional vertigo, through computational fluid dynamics analysis from magnetic resonance image reconstructions

**DOI:** 10.3389/fneur.2025.1561356

**Published:** 2025-03-11

**Authors:** Marcos Rossi-Izquierdo, Sofia Santos-Pérez, Ismael Arán-Tapia, Miguel Blanco-Ulla, Ismael Arán-González, Isabel Vaamonde-Sánchez-Andrade, Virginia Franco-Gutiérrez, Vicente Pérez-Muñuzuri, Alberto P. Muñuzuri, Andrés Soto-Varela

**Affiliations:** ^1^Department of Otorhinolaryngology, University Hospital Lucus Augusti, Lugo, Spain; ^2^Division of Neurotology, Department of Otorhinolaryngology, Complexo Hospitalario Universitario, Santiago de Compostela, Spain; ^3^Department of Surgery and Medical-Surgical Specialities, University of Santiago de Compostela, Santiago de Compostela, Spain; ^4^Health Research Institute of Santiago de Compostela (IDIS), Santiago de Compostela, Spain; ^5^Group of Non-Linear Physics, Department of Physics, Campus Sur, University of Santiago de Compostela, Santiago de Compostela, Spain; ^6^Cross-disciplinary Research Center in Environmental Technologies (CRETUS), University of Santiago de Compostela, Santiago de Compostela, Spain; ^7^Department of Radiology, Complexo Hospitalario Universitario, Santiago de Compostela, Spain; ^8^Department of Otorhinolaryngology, Complexo Hospitalario Universitario de Pontevedra, Pontevedra, Spain; ^9^Galician Center for Mathematical Research and Technology (CITMAga), Santiago de Compostela, Spain

**Keywords:** benign paroxysmal positional vertigo, computational fluid dynamics, MRI, canalith repositioning maneuvers, mechanical rotation chair

## Abstract

**Background:**

Benign paroxysmal positional vertigo (BPPV) is the most common cause of vertigo, often effectively treated with standard canalith repositioning maneuvers (CRMs). However, approximately 12.5% of cases remain refractory, leading to persistent symptoms and increased healthcare burden. Variations in the anatomical orientation of the semicircular canals (SCCs) may explain the resistance to conventional maneuvers. This study explores a personalized medicine approach, utilizing computational fluid dynamics (CFD) based on MRI reconstructions to tailor CRMs with the help of mechanical rotation chair according to individual inner ear anatomy.

**Methods:**

We conducted a randomized, multicenter, open-label study targeting patients with refractory posterior canal BPPV. Participants were allocated to either a control group (receiving repeated standard CRMs and Brandt-Daroff exercises) or an intervention group (receiving personalized CRMs based on CFD simulations derived from MRI scans). The intervention group’s maneuvers were executed using a mechanical rotational chair designed for precise angulation. Primary outcomes included resolution of nystagmus and vertigo symptoms, while secondary outcomes measured the reduction in healthcare visits and improved quality of life (Dizziness Handicap Inventory score).

**Discussion:**

Personalized CRMs based on CFD models may enhance treatment efficacy for refractory BPPV by optimizing maneuver angles according to the specific SCC orientation. This approach could significantly reduce symptom persistence, decrease the need for repeated healthcare visits, and improve patient outcomes. The use of non-invasive MRI and CFD techniques represents a novel step toward individualized treatment in vestibular disorders, with potential for broader application in personalized otoneurology. Further analysis will determine the extent of clinical benefit and cost-effectiveness of this approach.

**Clinical trial registration:**

ClinicalTrials.gov, Identifier: NCT06725966.

## 1 Introduction

Vertigo is a common symptom in the general population, significantly limiting the ability of affected individuals to perform many activities, even the most basic daily tasks (1). The most frequent cause of vertigo is benign paroxysmal positional vertigo (BPPV) (2), with an estimated incidence ranging from 10 to 140 per 100,000 people and a lifetime prevalence of 2.4% (3, 4).

BPPV is caused by the displacement of otoliths, which normally adhere to the maculae of the utricle and saccule. When these otoliths detach, they float freely in the endolymph of the posterior labyrinth and often enter one of the semicircular canals, most frequently the posterior canal (4), followed by the horizontal and superior canals. In most cases, the otoliths move freely within the canal (canalithiasis), shifting under the influence of gravity when the patient changes the position of their head. This movement generates an endolymphatic current that stimulates sensory cells in the ampulla, resulting in nystagmus and a subjective sensation of vertigo. These patients experience recurrent episodes of brief vertigo (less than one minute), typically triggered by head movements such as lying down, sitting up, turning in bed, bending forward, or looking upward.

Occasionally, otoliths may adhere to the cupula (cupulolithiasis) rather than moving freely in the canal. The clinical effect is similar to that of canalithiasis: certain head movements cause deflections in the ampulla, where increased density due to the adhered otoliths leads to repeated vertigo episodes.

Diagnosis is achieved through provocation maneuvers (5, 6), where specific head positions cause otolith movement in the semicircular canals, resulting in vertigo and nystagmus. The characteristics of the nystagmus (mainly its direction) help determine which canal is affected, and in the case of the horizontal canal, whether it involves canalithiasis (geotropic nystagmus) or cupulolithiasis (ageotropic nystagmus). The main diagnostic maneuvers include the Dix-Hallpike test (7), McClure test, and head extension. Although diagnosis can be made through direct observation of nystagmus with the naked eye or using amplification systems like Frenzel glasses, videonystagmography is ideal as it allows for quantifying nystagmus intensity and duration as well as determining its direction.

Treatment, similar to diagnosis, involves maneuvers aimed at repositioning the otoliths back into the vestibule, expelling them from the semicircular canals. Specific maneuvers are available for each canal: Epley (8) and Semont (9) for the posterior canal, Lempert (10) and Gufoni for the horizontal canal, and Yacovino (11) or reverse Epley for the superior canal. The effectiveness of these maneuvers is high, with resolution rates around 90% for the most commonly affected canal (the posterior canal). Traditionally, these maneuvers are performed manually on a treatment table, although this method does not guarantee precise angulation of movements. Recently, a mechanized chair has become available, allowing precise execution of each maneuver and facilitating treatment in patients with mobility issues (e.g., obesity or cervical spine stiffness).

Despite the high success rate of these maneuvers, a significant number of patients [estimated at 12.5% of BPPV diagnoses (12)] do not achieve resolution of symptoms. These cases result in repeated healthcare visits, prolonged periods of work disability, and significant family burden, turning an otherwise benign and easily treatable condition into a persistent medical, social, and occupational problem.

Various strategies have been proposed for the treatment of refractory BPPV. These include habituation exercises [such as Brandt-Daroff maneuvers (13), aimed at dispersing the otoliths], transtympanic corticosteroid instillation (14) before performing maneuvers (attempting to reduce potential edema in the membranous labyrinth that may prevent otolith movement toward the vestibule during repositioning), and even surgical interventions (e.g., canal plugging or sectioning of the singular nerve). However, none of these approaches have definitively resolved the issue, either due to the low efficacy of certain methods (e.g., Brandt-Daroff maneuvers or corticosteroid instillation) or the significant side effects associated with surgical interventions (e.g., hearing loss and intracranial complications).

One plausible explanation for the ineffectiveness of maneuvers in certain patients may be individual variations in the spatial arrangement of the semicircular canals. Typically, the canals form angles of approximately 90° relative to one another, and repositioning maneuvers are designed based on this anatomical assumption. However, it is well known that there are individual differences in the angles between these structures (15), potentially resulting in a mismatch between the patient’s anatomy and the movements used in standard maneuvers. A mathematical model indicating the exact anatomical arrangement of the semicircular canals and predicting the expected movement of otoliths would allow the maneuver to be tailored to the specific anatomy of each patient, essentially enabling personalized medicine.

For patients with refractory BPPV, imaging (specifically, magnetic resonance imaging of the posterior cranial fossa) is recommended to rule out a neurological cause (16). This MRI typically includes the internal ears of the patient. From a routine clinical MRI of the inner ear, a personalized mathematical model can be developed. This process involves several stages and has been successfully applied in the development of personalized therapies in other fields, such as Cardiology. Initially, the digital version of the MRI is used to create a 3D model representing the endolymphatic fluid container. From this, a mathematical model based on Navier–Stokes equations is developed and solved using Computational Fluid Dynamics (CFD) techniques.

CFD-based numerical simulations offer a non-invasive tool for analyzing the human vestibular system, capable of predicting experimentally observed behaviors. For example, they can estimate the transcupular pressure threshold below which the membranous labyrinth’s response ceases to be linear, known as the mechanical adaptation effect (17). This innovative approach provides insights into understanding various vestibular pathologies, such as the Tullio phenomenon (18) and Ménière’s disease (19).

**Figure 1:**
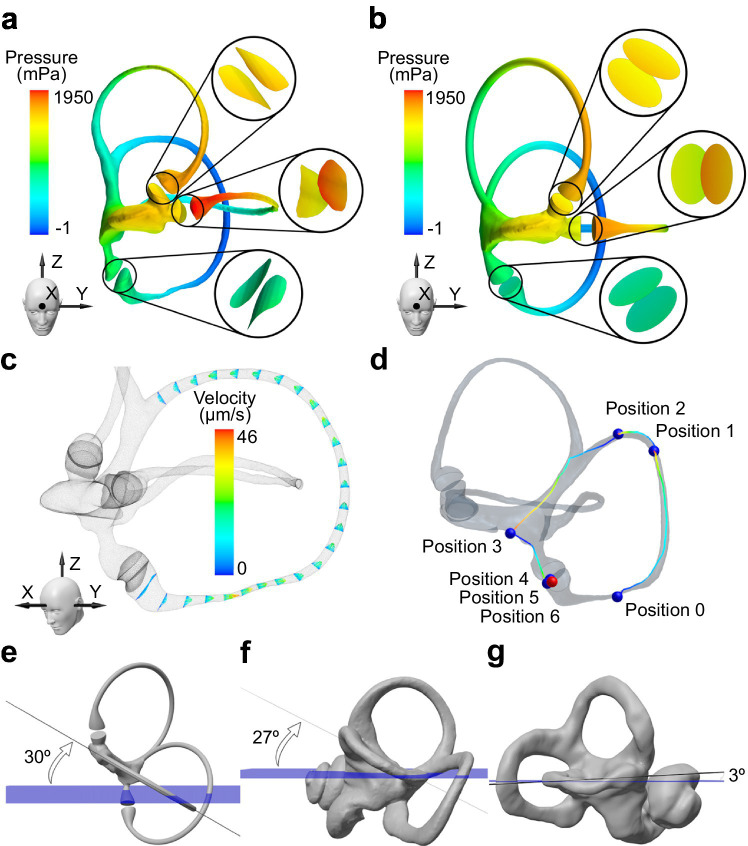
Visualization and analysis of key anatomical and physical parameters with implications for BPPV treatment. Pressure distribution on the walls of the membranous labyrinth during rotation around the Z-axis for **(a)** real and **(b)** ideally orthogonal semicircular canals. **(c)** Endolymphatic velocities in the posterior semicircular canal obtained during rotation around the Z-axis. A Poiseuille flow with an ampullifugal direction is observed. **(d)** Displacement of otoconia during the standard Epley maneuver. The otoconia exit the posterior semicircular canal but do not reach the utricular macula, indicating that the maneuver cannot be considered successful. Orientation of the horizontal semicircular canal relative to the Frankfurt plane (blue) for **(e)** an idealized model with orthogonal canals, **(f)** the bony labyrinth of the subject shown in the previous figures, and **(g)** the bony labyrinth of a patient refractory to standard treatment. The anomaly in the orientation of the semicircular canals may explain the ineffectiveness of the treatment.

Through numerical simulation and CFD analysis of a high-resolution micro-CT as described in previous work (20), we previously conducted a study to evaluate vestibular stimulation on the crista ampullaris during rotations mimicking the Head Impulse Test (HIT) (21). Instead, for this clinical study, we focused primarily on the pressures on the labyrinth walls and velocities of the endolymph when combined with a particle model representing the otoliths. Examples of the results obtained from these simulations are shown in Figures 1a–d.

Such models provide valuable insights into the underlying causes of BPPV and help optimize individualized treatment strategies for patients who do not respond to traditional maneuvers (22). For instance, Figures 1e–g highlight the significant deviation of the horizontal semicircular canal in a refractory patient compared to a healthy or idealized model. This anomaly may explain the ineffectiveness of the standard maneuvers for treating BPPV.

If the research hypothesis is correct, adjusting the head movement angles could tailor the repositioning maneuvers to each patient’s specific anatomy. This adaptation could be accurately implemented using a mechanized chair designed for high precision. This approach would enable faster symptom resolution, reduce healthcare costs (in terms of fewer repeated consultations), and shorten the duration of work and functional disability associated with persistent vertigo.

The main objective of the present study is to enhance the effectiveness in treating patients with refractory benign paroxysmal positional vertigo (BPPV) of the posterior semicircular canal through personalized otolith repositioning maneuvers (using a mechanized chair), tailored to the spatial arrangement of each patient’s semicircular canals. This will be compared with the repetition of conventional maneuvers and the use of Brandt-Daroff exercises.

The secondary objectives are:

- To determine whether there are differences at the time of diagnosis (mainly in the characteristics of nystagmus) between patients with refractory BPPV and those who respond well to conventional maneuvers.
- To develop software capable of generating a mathematical model based on MRI images of the patient’s inner ear, predicting the movement angles necessary to guide the otoliths back to the vestibule. This model will also provide additional information that can be used to rule out or confirm the presence of other underlying pathologies.
- To quantify the potential reduction in the subjective perception of disability achieved by performing modified, personalized maneuvers compared to the repetition of conventional maneuvers and Brandt-Daroff exercises.
- To quantify the reduction in healthcare resources (primarily in the number of consultations) achieved through treatment with personalized maneuvers.

## 2 Methods and analysis

### 2.1 Study design

This is an experimental, multicenter, open-label, randomized study (using balanced block randomization) with two parallel arms. The study targets patients with a definitive diagnosis of refractory posterior semicircular canal BPPV (BPPVp).

### 2.2 Study participants

*Study period*: From January 1, 2024, to December 31, 2026.

*Study population*: Patients diagnosed with BPPV.

*Target population*: Individuals >18 years of age, of both sexes, diagnosed with unilateral refractory BPPVp, from three Health Areas. Participants will be selected from patients attending the Otoneurology Unit of the Otolaryngology Departments of these three University Hospital Complexes.

*Inclusion criteria*: Participants must understand the study objectives, agree to participate, and sign the informed consent form. Candidates must meet the following two requirements:

- Diagnosed with BPPVp according to the Barany Society criteria:

- Recurrent episodes of vertigo or positional instability, triggered by lying down or turning the head in the supine position.
- Episodes lasting less than 1 min.
- Positional nystagmus observed after a latency period during the Dix-Hallpike test (DHT), with an upward vertical component and a torsional component (clockwise on the left side and counterclockwise on the right).
- Symptoms not explained by any other cause.

- Refractory to otolith repositioning maneuvers (Epley and/or Semont maneuvers), with persistence of symptoms after three attempts.

*Exclusion criteria*: Participants will be excluded if they have:

- Cognitive impairment that prevents understanding of their condition and the required procedures.
- Pathological conditions that hinder the execution of the maneuvers.
- BPPV involving canals other than the posterior canal or bilateral posterior canal involvement.
- Patients who fail to demonstrate two successful repetitions of Brandt-Daroff maneuvers when provided with one set of verbal instructions and once set of printed instructions.

To confirm the diagnosis and rule out other potential causes, all participants will undergo a complete otoneurological evaluation, including:

- Otoscopy.
- Basic neurological examination.
- Observation of spontaneous nystagmus (with and without gaze fixation).
- Clinical head impulse test (vHIT), including video-assisted evaluation.
- Positional tests (DHT, McClure test, and head hyperextension), with videonystagmographic recording.
- Pure-tone audiometry.

In cases of refractory BPPV (lack of resolution after three therapeutic maneuvers), a magnetic resonance imaging (MRI) scan of the inner ears and posterior cranial fossa will be requested to rule out central neurological causes.

### 2.3 Randomization

*Pre-selection visit (Visit − 1)*: Participants will undergo a comprehensive vestibular assessment to identify candidates with refractory BPPVp. The following tests will be included:

- Video-assisted head impulse test (vHIT) to assess for other vestibular pathologies.
- Dix-Hallpike test (DHT) to confirm the diagnosis of BPPVp.
- McClure test to exclude horizontal canal BPPV.
- Head hyperextension test to exclude superior canal BPPV.
- Dizziness Handicap Inventory (DHI) questionnaire adapted to Spanish, assessing perceived disability due to vertigo. Patients complete it independently at each participating hospital. This ensures consistency in subjective outcome reporting across study sites, minimizing potential biases introduced by different evaluators.

For posterior canal BPPV, it will be verified in the medical record that three repositioning maneuvers (Epley and/or Semont) were performed without symptom resolution.

Eligible participants will be given a detailed explanation of the study objectives and procedures, and will receive the approved Patient Information Sheet (PIS) to provide informed consent.

### 2.4 Randomization process

*Visit 0 (Randomization visit)*: After obtaining consent, participants will be randomized to one of the following study arms:

- Control group: Participants will be advised to perform Brandt-Daroff exercises at home twice daily for 8 weeks. An MRI of the inner ear and posterior cranial fossa will also be requested. If the exercises are ineffective, repositioning maneuvers will be repeated biweekly until symptom resolution using a mechanical rotational chair, specifically the Thomas Richard Vitton (TRV) reposition chair.
- Study group: Participants will perform Brandt-Daroff exercises at home twice daily for 8 weeks. An MRI will be conducted, and based on the images, a personalized mathematical model will be created to modify the angles of the repositioning maneuver. If the exercises are ineffective, the personalized maneuver will be performed using a mechanized chair.

A balanced block randomization sequence with block size n = 10 will be generated by a member of the research team.

### 2.5 Study variables

The study variables are as follows:

- Age at inclusion.
- Sex.
- Duration of symptoms (in months).
- Etiology of BPPV.
- Affected ear.
- Nystagmus latency during the Dix-Hallpike Test (DHT) (in seconds).
- Nystagmus duration during the DHT (in seconds).
- Mean slow-phase velocity of the vertical component of nystagmus during the DHT (in degrees/s).
- Dizziness Handicap Inventory (DHI) score at inclusion and 6 months after inclusion.
- Resolution of symptoms after treatment (yes or no).
- Resolution of nystagmus (PRIMARY VARIABLE) after treatment (yes or no).
- Number of consultations required for resolution (to quantify the reduction in healthcare resources).

### 2.6 Data collection

Even if intermediate visits are necessary, the following study visits will be conducted according to the protocol (with data recording):

Visit 1 (eight weeks after inclusion):

- The Dix-Hallpike Test (DHT) will be performed with videonystagmographic recording. If positive:
- In the control group, the conventional Epley maneuver will be repeated using a mechanized chair.
- In the study group, a personalized Epley maneuver will be performed using a mechanized chair.

Visit 2 (ten weeks after inclusion):

- The DHT will be repeated with videonystagmographic recording. If positive:
- In the control group, the conventional Epley maneuver will be repeated using a mechanized chair.
- In the study group, the personalized Epley maneuver will be repeated using a mechanized chair.
  These maneuvers (conventional for the control group and personalized for the study group) will be repeated every two weeks until resolution of symptoms (or up to a maximum of 8 repetitions).

Visit 3 (Six months after inclusion):

- The following variables will be recorded:
- Dix-Hallpike Test (DHT) results (negative or positive).
- Dizziness Handicap Inventory (DHI) score.
- Number of consultations required for resolution.

### 2.7 Mathematical study

A 3D model of the affected membranous labyrinth will be reconstructed based on MRI imaging. An analysis of the planes on which the semicircular canals rest will be performed, and the geometric model will be adapted for subsequent numerical simulation.

The primary requirement of the MRI is that the scan must be a volumetric sequence (with very thin slices and no gaps between them), fluid-sensitive (T2-weighted), and must include the eyeballs within the field of view to provide a reference for identifying the Frankfurt plane.

The computational study, carried out using Computational Fluid Dynamics (CFD) techniques, will involve combining the dynamics of the endolymph within the labyrinth and the particle dynamics of the free otoliths.

Mathematically, the Navier–Stokes equations will be coupled with a discrete particle model sensitive to gravity and the fluid forces. This will allow for the determination of the velocity distribution of the endolymph, the pressures exerted on the labyrinth walls, as well as the timing and positions of the otoliths during the personalized Epley maneuver.

Notably, the simulations were performed using Star CCM+, a widely recognized computational fluid dynamics (CFD) solver used in both research and industry. While other tools exist for real-time “simulation,” in our opinion, they lack the precision required for detailed biomechanics and particle-fluid interactions, which are critical for accurately modeling otoconial displacement.

The primary objective of the fluid simulation is to optimize the maneuver angles for treating BPPV. While canal alignment is an important factor, the simulation extends beyond anatomical variations by also considering fluid dynamics and otolith behavior during repositioning maneuvers.

In other words, successful BPPV treatment is not solely determined by canal orientation but rather by the complex interplay of forces acting on the fluid and otoconia. The CFD model allows us to predict how otoconia move within the semicircular canals under different maneuver conditions, enabling us to tailor the maneuver angles accordingly.

We expect that this more accurate approach, combined with the precise anatomical data of each refractory patient, will enhance treatment outcomes.

The final version of our mathematical model represents a balance between computational cost and accuracy, ensuring precise simulation of fluid and otoconia dynamics. For instance, large displacements—such as those involving full-body rotations from the waist—introduce inertial forces that can alter the trajectory of otoconia. This effect can lead to different final otoconia positions after 30 s of resting time, which in turn impacts the effectiveness of repositioning maneuvers.

Our analysis considers variations of 9° in the excursion angle of both rotational arms of the mechanical chair. We consider that the error margin inherent in these models is contained within this range, as previous simulations showed that variations in different parameters resulted in deviations smaller than this threshold.

To identify the optimal maneuver, we simulate different otoconial sizes and select the configuration that facilitates the greatest otoconial displacement toward the exit of the posterior canal. In the final steps—once the otoconia exit the canal—we ensure their translation toward the utricular macula for complete repositioning.

By incorporating these factors, our CFD-based approach enables a more precise and individualized treatment strategy for refractory BPPV cases.

We ensured reproducibility by conducting repeated simulations using the same patient’s vestibular anatomy. We ran hundreds of simulations, testing different solvers, meshes, boundary conditions, and other parameters related to vestibular physiology. Based on this analysis, we concluded that our model has certain limitations, such as assumptions regarding particle shape, particle interactions, and wall conditions, among others. These limitations and more information about this mathematical model, designed for studying BPPV treatment, are discussed in detail in our previous study (22).

### 2.8 Data analysis

Demographic data and patient characteristics will be described globally and by relevant covariables.

Continuous outcomes will be summarized using mean, median, standard deviation (SD), and interquartile range (P25, P75). For categorical outcomes, the number and percentage of patients in each category will be reported. Statistical analyses will be based on the full analysis set (FAS), which includes all randomized participants who provided informed consent and met all inclusion criteria without any exclusion criteria. The analysis will follow the intention-to-treat principle.

The key outcome variables of interest are:

- Resolution of nystagmus (yes vs. no), which is the primary outcome.
- Resolution of symptoms (yes vs. no).
- DHI score (minimum: 0; maximum: 100) at inclusion and 6 months after inclusion.
- Number of consultations required until resolution.

Covariables of interest include:

- Baseline patient characteristics:
- Sex.
- Age at inclusion.
- Nystagmus latency period during the Dix-Hallpike Test (in seconds).
- Nystagmus duration during the Dix-Hallpike Test (in seconds).
- Mean slow-phase velocity of the vertical component of nystagmus during the Dix-Hallpike Test (in degrees/s).
- Associated risk factors:
- Duration of symptoms.
- Etiology.
- Adherence to the intervention (direct observation by the investigator in both intervention arms).

### 2.9 Study limitations, data biases, and mitigation strategies

Despite being an open-label study, the potential bias introduced is minimal, as both the maneuvers and outcome evaluation are performed by the same person. The primary outcome (resolution of nystagmus) is assessed objectively through videonystagmography, which does not require interpretation by the evaluator.

There is a risk of patient dropout during the eight-week period of Brandt-Daroff exercises prior to the personalized maneuver. To mitigate this risk, patient adherence will be closely monitored and encouraged with reminders and follow-ups.

Since patients will be recruited from three different hospitals, there may be diagnostic biases depending on the evaluator. However, all intervention maneuvers (both personalized and conventional) will be conducted at a single center, where a diagnostic reevaluation will be performed prior to intervention to confirm that the BPPV remains unresolved and exclusively affects the posterior canal.

### 2.10 Sample size and calculation methodology

To estimate the sample size, we assume that approximately 25% of patients with refractory BPPVp may be cured through repeated maneuvers or Brandt-Daroff exercises (control group). A significant difference of 50% in the study group (i.e., a 75% efficacy rate for symptom resolution) is considered clinically meaningful. With a 95% confidence level (1-*α*) and 95% statistical power for a two-tailed hypothesis test, a sample size of 23 participants per arm is required. Thus, the estimated total sample size is 46 individuals, increased to 54 (27 per group) to account for a 15% anticipated dropout rate.

## 3 Discussion

Previous studies have compared the effectiveness of manual canalith repositioning maneuvers (CRMs) against the TRV chair in patients diagnosed with BPPV, focusing on treatment success, quality of life, and recurrence rates. However, while mechanical chairs can be useful, especially in specific subpopulations (elderly patients with mobility limitations), they do not significantly outperform manual maneuvers (23).

Another recent study has shown that TRV chair could be useful in diagnosis for complex BPPV cases, patients with impaired cooperation or as a second opinion diagnostic tool for treatment-resistant BPPV (24).

Specifically, a systemic review focused on the use of multi-axial repositioning chairs (25), like the TRV chair and Epley Omniax Rotator, in the diagnosis and treatment of BPPV have found:

- Symptom relief was high, with treatment success rates between 68 and 100%.
- Recurrence rates ranged from 11 to 27.9%.
- Multi-axial chairs were found effective, particularly for patients who could not undergo manual repositioning or had rarer forms of BPPV.
- Minor adverse effects like nausea and claustrophobia were reported in some cases.

So, the conclusion drawn was that Repositioning chairs appear safe and effective for managing BPPV, including difficult cases. However, the lack of RCTs limits the ability to compare their effectiveness directly with traditional manual methods.

Recently, a study has aimed to investigate whether patients with very refractory BPPV could be effectively treated using a mechanical rotational chair (MRC) tailored to individual inner ear anatomy (26). The MRC allows for individualized adjustments based on specific angles of the semicircular canals (SCC), and the inner ear anatomy was evaluated using CT scans. Complete remission of both symptoms and objective findings was achieved in 21.4% of patients, and subjective relief was reported in 42.9% of patients. Noteworthy, the study found significant differences in SCC angles from standard anatomical assumptions, which may explain the refractoriness to traditional treatments.

Our current study protocol is based on MRI imaging (to avoid x-ray) to measure individual SCC-angles and latter prepare individual treatments adjustable with the MRC.

To the best of our knowledge, this is the first study evaluating the possibility of resolving refractory BPPVp through the customization of maneuver angles with the application of CFD techniques. The study is relevant as it aims to adapt existing technologies (MRI, CFD techniques, and the TRV mechanized chair) to achieve cost savings and improve the quality of life for these patients.

In future studies, we plan to personalize the resting time based on specific nystagmus characteristics or patient conditions. Additionally, parameters such as rotation velocity may be explored to further optimize certain maneuvers.

Also, future studies should integrate experimental validation with patient-specific vestibular assessments.

The use of CFD modeling based on MRI reconstructions represents a novel step toward personalized medicine in vestibular disorders. While this study focuses on refractory BPPV, the approach could be extended to other vestibular conditions where individualized anatomical variability plays a role in treatment efficacy. Future studies should investigate the integration of these methods to optimize vestibular disorder management and enhance treatment outcomes across different patient populations.

Additionally, the project will have an incentivizing effect on the institution: the application of CFD techniques to the study of endolymphatic fluid movement opens the door to new research lines aimed at optimizing diagnostic tests for vestibular function.

## 4 Ethics and dissemination

The data will be collected in a specially designed database. There will be periodic data quality controls. The PI will monitor the study and ensure that the data are authentic, accurate and complete, and that the subjects’ safety and rights are protected.

The protocol has been approved by the Independent Ethics Committee of Galicia (protocol 2022/143).

The study will be carried out in compliance with current ethical standards and in accordance with the principles established in the Declaration of Helsinki, including all applicable amendments set forth by the ICH guidelines for Good Clinical Practice. Similarly, it will be ensured that all individuals involved in the project respect the confidentiality and security of any information related to the study subjects, in accordance with the provisions of Law 3/2018 on Personal Data Protection and Guarantee of Digital Rights. Upon completion of the study, the results will be disseminated and published, taking into account that the data will be anonymized, coded, and de-identified in order to deposit them in the designated repositories and enable their use in future research.
